# An Important Decision

**Published:** 1884-07

**Authors:** 


### AN IMPORTANT DECISION.

In this number will be found a very important legal decision in which the Supreme Court of the State of Illinois affirms the authority of State Dental Societies, or Examining Boards, to determine what are "reputable dental colleges" whose diplomas may be recognized as authority and license for the practice of dentistry. Application was made to the court for a mandamus, compelling the Dental Examining Board of the State of Illinois to recognize a diploma granted by the Indiana Dental College. The decision affirms the fact that in making this determination the Board acts in a judicial capacity, and that from its action there is no appeal. It is evident that had the decision been otherwise, there would be no check whatever upon colleges, and they would have been able entirely to control and dominate the profession, and admit to practice whom they pleased. Wisconsin colleges might have multiplied, and we should be forced to acknowledge every fraudulent diploma issued.

